# Evaluation of a Fish Gelatin-Based Edible Film Incorporated with *Ficus carica* L. Leaf Extract as Active Packaging

**DOI:** 10.3390/gels9110918

**Published:** 2023-11-20

**Authors:** Hanan Rizqy Fauzan, Andriati Ningrum, Supriyadi Supriyadi

**Affiliations:** Department of Food and Agricultural Product Technology, Faculty of Agricultural Technology, Universitas Gadjah Mada, Flora Street No. 1, Bulaksumur, Yogyakarta 55281, Indonesia

**Keywords:** edible film, fish gelatin, sustainable packaging, *Ficus carica* L.

## Abstract

The significant concerns associated with the widespread use of petroleum-based plastic materials have prompted substantial research on and development of active food packaging materials. Even though fish gelatin-based films are appealing as active food packaging materials, they present practical production challenges. Therefore, this study aimed to develop an edible film using *Ficus carica* L. leaf extract (FLE), as it is affordable, accessible, and has superoxide anion radical scavenging action. This edible film was produced by adding FLE to mackerel skin gelatin at varied concentrations (2.5–10% *w*/*w*). The results showed that adding FLE to gelatin films significantly affected the tensile strength (TS), elongation at break (EAB), transmittance and transparency, solubility, water vapor permeability (WVP), antioxidant activity, and antibacterial activity. Among all the samples, the most promising result was obtained for the edible film with FLE 10%, resulting in TS, EAB, solubility, WVP, antioxidant activity, and antibacterial activity against *S. aureus* and *E. coli* results of 2.74 MPa, 372.82%, 36.20%, 3.96 × 10^−11^ g/msPa, 45.49%, 27.27 mm, and 25.10 mm, respectively. The study’s overall findings showed that fish gelatin-based films incorporated with FLE are promising eco-friendly, biodegradable, and sustainable active packaging materials.

## 1. Introduction

Packaging is essential for maintaining the quality of food products by protecting them from all forms of physical, chemical, and microbiological damage [1]. The most common material used in food packaging comes from petroleum. The packaging industry prefers these materials because they form highly transparent films and have mechanical strength, water vapor resistance, and gas resistance [2]. However, if not treated properly in the process, these films can cause environmental pollution. Most petroleum-based plastics are not biodegradable and can break down into dangerous microplastics, threatening ecosystems and wildlife. Furthermore, improper disposal of plastic packaging can result in litter accumulating in natural environments and contributing to plastic pollution in oceans and rivers. Therefore, it is necessary to develop an alternative food packaging film made from natural, biodegradable, and sustainable materials [3,4].

The use of biodegradable biopolymers for food packaging has gained interest, given the inconvenience to sustainability of conventional food packaging [5]. Biodegradable and renewable polymers, including proteins, can replace non-environmentally friendly synthetic packaging. Proteins have the potential to form transparent films with specific mechanical properties, excellent oxygen barrier capabilities, and the ability to form tissues and induce plasticity and elasticity [6,7]. Gelatin is one form of protein that may be utilized to generate edible films. Gelatin is a denatured protein with exceptional gelling characteristics and is commonly used in the food industry. Gelatin-based edible films have potent gas and lipid barrier properties and can incorporate antibacterial or antioxidant compounds [6,7].

Globally, most gelatin comes from the skin and bones of land mammals [8]. However, gelatin derived from land mammals can cause bovine spongiform encephalopathy (BSE) and foot and mouth disease (FMD), so its safety is questionable. Even foods and medicines with a gelatin composition derived from land mammals have been banned by several European countries [9]. Gelatin derived from the skin of narrow-barred Spanish mackerel (*Scomberomorus commerson*) can be an alternative because the gelatin content is relatively high, at around 18% [10]. In Indonesia, mackerel production in 2020 reached 435,835.39 tons. This vast amount of production will undoubtedly result in a substantial amount of fish skin by-products produced by the fish processing industry [11].

Gelatin-based films can be combined with plant extracts with bioactive compounds that enhance the functional properties of the films. Many edible films have been developed by incorporating plant extracts, such as green tea extract (GTE) and *Lepidium sativum* extract (LSE), into gelatin. A higher GTE content integrated into gelatin films confers significantly higher tensile strength and lower elongation at break, water solubility, and water vapor permeability. Moreover, there is no notable difference in the mechanical parameters of tensile strength and elongation at break in films enriched with LSE [12,13].

*Ficus carica* L., commonly known as fig, originated in western Asia (Turkey), and its cultivation spread to other parts of the world through Mediterranean countries. This plant belongs to the Moraceae family and is believed to have been used as a medicinal herb for centuries. The fruit, roots, and leaves of the *Ficus carica* L. plant contain phytochemicals and various bioactive compounds. Specifically, the leaves are rich in polyphenols with antioxidant, anticancer, antidiabetic, and anti-inflammatory properties and hepatoprotective activity that could benefit human health [14,15,16,17]. Therefore, the leaf extracts of this plant are suitable for incorporation into gelatin to form an edible film. Fig leaf extract (FLE) contains flavonoids, terpenoids, and tannins that can enhance the physical properties of gelatin edible films and increase their antioxidant activity [18,19]. Nevertheless, no study has reported the incorporation of FLE into fish gelatin as an active food packaging material. Hence, this study was carried out to assess the physical properties, antioxidant activity, and antimicrobial activity of mackerel skin gelatin-based films enriched with fig leaf extract (FLE) as a bioactive component in these films to create eco-friendly, biodegradable, and sustainable active packaging. 

## 2. Results and Discussion

### 2.1. Fig Leaf Extract (FLE) Antioxidant Activity

Before being used for film preparation, fig leaf extract was tested for its antioxidant activity using the DPPH method. From the tests that were conducted, fig leaf extract requires a concentration of 27.18 ± 0.44 ppm to inhibit 50% of DPPH free radicals. In comparison, BHT requires 19.75 ± 0.23 ppm. These findings demonstrated that fig leaf flavonoids had high antioxidant activity. Flavonoids and polyphenols have a higher concentration of phenolic hydroxyl groups, which exert antioxidant action by lowering hydroxyl groups. They can help stabilize free radicals and act as antioxidants by supplying hydrogen ions [20].

### 2.2. Color

**Figure 1:**
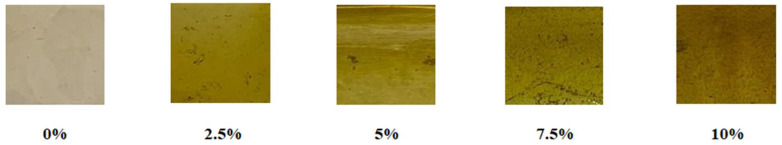
Development of an edible film enriched with *Ficus carica* L. leaf extract.

As FLE was incorporated into fish gelatin films, the color of the films changed from greenish to yellowish, and this finding was validated by color parameter measurements. As shown in Table 1, increasing FLE incorporation into fish gelatin films raised the b and ∆E values of the films while decreasing the L and a values. The ΔE measurement quantifies the difference between the displayed color and the original color standard of the input content. The results were consistent with a prior study, which found an increase in yellowness after incorporating plant extract into gelatin-based films [7,21]. Previous research also showed that increasing the concentration of fig leaf extract causes a decrease in brightness and redness but an increase in yellowness and ΔE in chitosan-based films [7,22]. The observed color variations in the film were generated by naturally colored pigments in the *Ficus carica* L. extract (Figure 1).

### 2.3. Transmittance and Transparency

**Figure 2:**
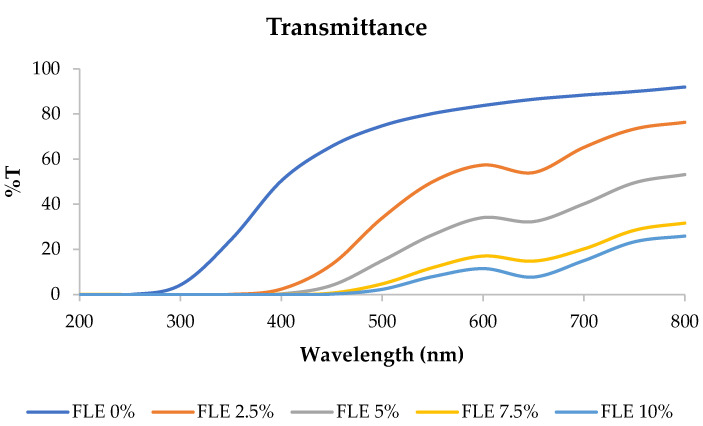
Transmittance of fish gelatin edible films incorporated with *Ficus carica* L. leaf extract.

Transmittance refers to the ability of an edible film to allow the passage of light or other electromagnetic radiation through it. Films with high transmittance are more transparent, allowing more light to pass through, while those with low transmittance are less transparent or even opaque [7]. The transmittance of fish gelatin edible films incorporated with FLE at 200–800 nm wavelength is shown in Figure 2. The percentage of light that could pass through the edible film decreased as the concentration of FLE increased.

These results determined the transparency value of the film, where the transparency value was directly proportional to the film’s transmittance. The transparency values are presented in Table 1. Increasing the concentration of FLE significantly affected the transparency of the film samples. Higher transparency values correspond to a higher visible light absorbance, which indicates a lower transparency of the film [23,24,25,26,27,28,29,30,31,32,33]. Therefore, the edible film with 10% FLE had the best UV barrier but had the lowest transparency. 

This finding was in line with previous studies showing that phenolic compounds distributed within a film matrix affect the morphology and light transmission of films [34,35,36]. The edible film based on fish skin gelatin incorporated with FLE exhibited good UV barrier properties, making it suitable for application in products vulnerable to UV light-induced damage, especially foods susceptible to UV light-induced lipid oxidation [37,38].

### 2.4. Thickness

Table 2 shows the thickness of the gelatin films containing various concentrations of FLE. The addition of *Ficus carica* L. leaf extract to gelatin films resulted in no significant variation in film thickness. FLE was equally distributed in the gap between the gelatin film matrix because the hydroxyl groups of polyphenols in FLE generated intermolecular hydrogen interactions with the amino/hydroxyl groups in gelatin. As a consequence, the integration of FLE had no significant effect on the thickness of the gelatin films [23].

### 2.5. Tensile Strength, Elongation at Break, and Elastic Modulus

The effect of FLE inclusion in gelatin films on tensile strength (MPa), elongation at break (%), and elastic modulus was studied. Tensile strength (TS) is directly related to polymer chain cohesion, whereas elongation at break (EAB) is related to flexibility and extensibility before breaking. Moreover, the elastic modulus is a quantity that measures an object or substance’s resistance to being deformed elastically when a stress is applied to it. The interaction of the gelatin polymer chain with other components, including antioxidant extracts, water, and plasticizers, can have a substantial impact on the mechanical characteristics of the resultant mix films. Furthermore, the combination of the biopolymer matrix, plasticizer, and active agent might affect the mechanical characteristics of the resultant active blend films [24].

Table 2 shows the tensile strength (MPa), elongation at break (%), and elastic modulus (MPa) data. The TS of the gelatin-based films rose as the FLE concentration increased. This was due to the ability of hydroxyl groups in polyphenolic chemicals to form hydrogen bonds with hydrogen acceptors in gelatin molecules. The formation of intermolecular hydrogen bonds between FLE and gelatin rendered the films more compact and resistant to tensile stress [25]. A previous study discovered that including green tea extract raised the TS of gelatin films, which was induced by interactions between the phenolic compounds of the extract and the amino acids of the gelatin [26]. The addition of FLE, on the other hand, enhanced the EAB of the gelatin-based films. The slightly higher EAB in these gelatin-based films was most likely due to polyphenols acting as plasticizers, increasing the flexibility of FG-HBE films [27]. When plant extract was introduced into gelatin films, the EAB improved similarly because the interactions between polyphenols and gelatin created a more cohesive and flexible film matrix [28]. The addition of FLE increased the rigidity of the film, thus increasing the elastic modulus. This result is in line with similar research that demonstrated an increase in Young’s modulus as tensile strength values increased [29].

### 2.6. Solubility

Water solubility is defined as the proportion of water-soluble compounds in the film and is sometimes referred to as a film’s water resistance. According to the results, the water solubility of the films decreased dramatically in proportion to the FLE content (Table 3). These findings are consistent with prior studies in which green tea extract was combined with gelatin films [22]. With the addition of FLE, the gelatin films’ solubility decreased due to the creation of stronger cross-links between polyphenols and gelatin in the polymer matrix, which reduced the polar groups and hydrophilic qualities of the films [30].

### 2.7. Water Vapor Permeability (WVP)

WVP is a critical feature of edible films used in food packaging applications to protect food from water-induced deterioration. As demonstrated in Table 3, the WVP of FLE-gelatin films decreases steadily as the FLE content increases. The lower WVP values of all film formulations might be attributed to the formation of a film matrix via the interaction of phenolic chemicals with gelatin. The addition of fig leaf extract might limit the free volume in the polymer matrix and enhance the tortuosity of the water molecules’ passage across the network, lowering the rate of water molecule diffusion through the films [31]. According to the literature, comparable behavior was reported for green tea extract-gelatin films [22].

### 2.8. Antibacterial Activity of the Films

The antimicrobial activities of films against Gram-negative and Gram-positive bacterial species were evaluated by determining the inhibition zones (mm) on solid medium. Table 4 shows that the inhibition zone of the film against *Staphylococcus aureus* and *Escherichia coli* increased in size as the FLE concentration increased. A larger zone of inhibition indicates greater antibacterial activity. These findings are in line with similar research describing the antibacterial ability of gelatin-based films incorporated with *Ginkgo biloba* extract [39].

Previous studies have shown the antimicrobial activity of *Ficus carica* L. extracts against Gram-positive and Gram-negative bacteria. This antibacterial activity is due to phenolic compounds in FLE, such as flavonoids, caftaric acid, quercetin, p-hydroxybenzoic acid, caffeic acid, and gallic acid. Phenolic compounds can disrupt the integrity of bacterial walls and cell membranes, resulting in the release of intracellular components from microbial cells. This process inhibits microorganism growth by blocking electron transfer, nutrient absorption, nucleotide synthesis, and membrane ATP activity [39,40,41].

### 2.9. Antioxidant Activity of the Films

Because antioxidant films may preserve food from oxidation and deterioration, they are essential for active packaging. The DPPH radical scavenging experiment assessed the antioxidant properties of all the film samples.

**Figure 3:**
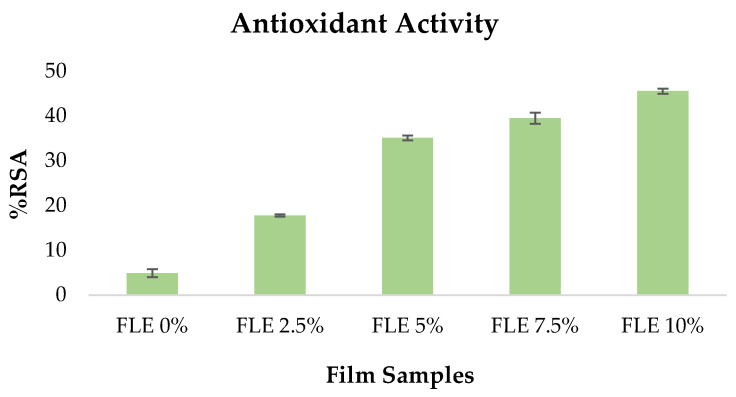
Antioxidant activity of fish gelatin edible films incorporated with *Ficus carica* L. leaf extract.

As demonstrated in Figure 3, integrating FLE greatly enhanced the DPPH value of the films. The powerful antioxidant capacity of phenolic compounds in FLE was ascribed to the increased DPPH radical scavenging activity in FLE-gelatin films. The phenolic components in *Ficus carica* L. extract chemically cross-link with the gelatin compound via covalent bonds. The oxidation of phenolic compounds to radicals results in covalent bonds between phenolic chemicals and proteins. These interactions between phenolic chemicals and gelatin amino acids enhance the antioxidant properties of the films [30].

## 3. Conclusions

In this study, fig leaf extract (FLE) was incorporated into gelatin-based edible films to develop a sustainable active packaging material. The results lead to the conclusion that the incorporation of FLE improved the properties of these edible films compared to control films. The edible film enriched with 10% FLE showed the best tensile strength (2.47 MPa), elongation at break (372.82%), solubility (36.2%), WVP (3.96 × 10^−11^ g/msPa), antioxidant activity (45.49%), and antibacterial activity against *S. aureus* (27.27 mm) and *E. coli* (25.10 mm). These findings align with previous studies indicating that the addition of plant extracts can enhance the characteristics of gelatin-based edible films. We hope that this study promotes the development of sustainable packaging that can be applied to several food matrices. A further physicochemical investigation is required for such novel gelatin-based films to study possible relaxations of the gelatin matrix with extract molecules. This investigation represents a part of a future project involving the application of such films as active food packaging materials for preservation.

## 4. Materials and Methods

### 4.1. Materials

Fish skin gelatin was obtained from the domestic industry in Bandung, Indonesia. Fig leaves (*Ficus carica* L.) were harvested at Jogja Ara Garden in Sleman, Indonesia. CV. Nura Jaya, Surabaya, Indonesia, provided food-grade glycerol. All chemicals were analytical grade.

### 4.2. Preparation of Fig Leaf Extract (FLE)

Fig leaves were cleaned and dried in a cabinet dryer at 50 °C for 24 h. Dried fig leaves were crushed and passed through a sieve with a mesh size of 60. For 48 h, 75 g of fig leaf powder was macerated in 600 cc of 70% ethanol (Merck, Germany) (1:8 *w*/*v*). The solution was filtered using Whatman No. 1 filter paper and concentrated at 40 °C in a rotating vacuum evaporator.

### 4.3. Antioxidant Activity of FLE

The antioxidant activity of the extract was determined using a previously described DPPH radical scavenging assay with modifications [6]. A total of 4 mg of DPPH was dissolved in 100 mL of methanol to make a 0.1 mM DPPH solution. Dissolving 0.1 g of FLE in 100 mL of methanol yielded a 1000 ppm stock solution. Following that, sample test solutions with graded concentrations of 10, 20, 30, 40, 50, and 60 ppm were created. One milliliter of each sample solution concentration was combined with two milliliters of a 0.1 mM DPPH solution. The mixture was vortexed and incubated for 30 min at 27 °C. A UV-VIS spectrophotometer was used to detect absorbance at 517 nm, with methanol serving as the blank solution. Using the following equation, the antioxidant ability (%Radical Scavenging Activity/%RSA) to inhibit free radicals was obtained:

(1) $$\%RSA=\frac{abs\;blanko-abs\;sample}{abs\;blanko}\times 100\%$$

The % inhibition of free radicals was plotted against the concentration of the test solution. The linear regression equation was used to get the IC_50_ value, which is the concentration of the test solution capable of blocking 50% of free radicals. Using the following formula, the computed results were inserted into the linear equation:

(2) y=ax+b

where *y* is the % inhibition (50), *a* is the slope, *x* represents the concentration (µg/mL), and b represents the constant.

### 4.4. Preparation of Edible Films from Mackerel Skin Gelatin Incorporated with Fig Leaf Extract

The preparation of gelatin edible films was based on a previous method with modifications [6]. At 50 °C, gelatin powder was dissolved in distilled water at a concentration of 4% (*w*/*v*) and agitated for 30 min at 500 rpm. The gelatin solution was then mixed for 5 min with 25% (*v*/*w*) glycerol from the quantity of gelatin. Furthermore, fig leaf extract in the amounts of 0%, 2.5%, 5%, 7.5%, and 10% (*w*/*w*) of the amount of gelatin was added to the mixture and mixed for 30 min. For 5 min, the solution was homogenized with an Ultra-Turrax homogenizer at 4000 rpm. The liquid was put into the mold and dried for 20–24 h in a cabinet dryer set to 50 °C, after which the edible film sheet was removed from the mold.

### 4.5. Film Characterization

#### 4.5.1. Color

The color analysis of the edible film was based on the previously reported method [6]. The color of the edible film was assessed using a digital colorimeter at six random spots on each sample with an 8 mm aperture, yielding values of L* (brightness), a* (red/green), and b* (yellow/blue). The total color difference (ΔE*) was calculated using the following equation:

(3) $$∆E*=\sqrt{{\left(L*-L_{0}*\right)}^{2}+{\left(a*-a_{0}*\right)}^{2}+{\left(b*-{\;b}_{0}*\right)}^{2}}$$

L*, a*, and b* are the color parameters of films incorporated with FLE; *L*_0_*, *a*_0_*, and *b*_0_* are the color parameters of the control films (100% gelatin films).

#### 4.5.2. Transmittance and Transparency

The transmittance and transparency of the films were measured according to a previously reported method [7]. The barrier qualities of the film against ultraviolet (UV) and visible light were tested using a UV-Vis spectrophotometer at the specified wavelength range of 200–800 nm, while the transparency value of the film was computed using the following equation:

(4) $$Transparency=2-{log}_{10}(\%T)/x$$

%*T* denotes the percent of transmittance at 600 nm, while *x* denotes the film thickness (mm).

#### 4.5.3. Thickness

The determination of film thickness was based on a previously reported method [7]. Ten spots on each sample were randomly measured with a micrometer, and the average value was calculated as the edible film thickness.

#### 4.5.4. Tensile Strength, Elongation at Break, and Elastic Modulus

Tensile strength (TS) and elongation at break (EAB) were determined using a previously reported approach and a Universal Testing Machine (UTM) [7]. Film samples were clipped to be 5 cm long and 1.5 cm wide. The film was put on the UTM stretcher and dragged by the device. With three replications, the test results yielded Fmax data, which are a measure of TS (MPa) and EAB (%). The elastic modulus (Young’s modulus) was obtained from the comparison of TS and EAB [32], expressed in mega Pascals (MPa), and formulated with the following equation:

(5) $$E=\frac{Tensile\;strength\;(MPa)}{Elongation\;at\;break}$$

#### 4.5.5. Solubility

The solubility of the edible films was determined using a previously described procedure [7]. Briefly, 2 × 2 cm samples were dried in an oven at 105 °C for 24 h. *W*_1_ was calculated after weighing the dry samples. Following that, the samples were immersed in 30 mL of aquades for 24 h at ambient temperature (25 °C). The samples were then dried again in a 105 °C oven for 24 h before being stored in a desiccator for 15 min. The samples were weighed, and *W*_2_ was obtained. The percentage of sample solubility in water was determined using the following equation:

(6) $$\%S=\frac{W_{1}-W_{2}}{W_{1}}\;\times \;100\%$$

*S* denotes the sample’s solubility, *W*_1_ the weight of the dried sample before immersion, and *W*_2_ the weight of the dried sample after immersion.

#### 4.5.6. Water Vapor Permeability (WVP)

The water vapor permeability of the film was calculated as previously described [7]. A glass cup-shaped container containing 6 mL of distilled water was sealed with the film at the mouth. The sample was then placed in a desiccator for 7 h at room temperature. Every hour, the sample was weighed using an analytical balance. The following equation was used to compute the *WVP* value:

(7) $$WVTR=\frac{∆W}{A\;∆P}$$

(8) $$WVP=\frac{WVTR\times L}{∆P}$$

where:

*WVTR* = the water vapor transmission rate;
Δ*W* = the weight of water vapor passing through the film (g);
*A* = the surface area of the film π.r^2^ (m^2^);
Δ*P* = the partial pressure difference in water vapor (Pa);
*L* = the thickness of the film (mm);
*WVP* = the water vapor permeability (g/msPa).

#### 4.5.7. Antibacterial Activity of the Edible Films

The antibacterial activity of the films was assessed against *Escherichia coli* (*E. coli*) and *Staphylococcus aureus* (*S. aureus*) using the method of a previous study with a slight modification [33]. The bacteria were individually cultured by transferring frozen *E. coli* and *S. aureus* into separate tubes of 10 mL of sterile nutrient broth with an inoculating loop. The organisms were incubated for 24 h at 37 °C for reactivation. Rejuvenation of the bacteria was carried out by streaking a loopful of pure culture onto a sterile nutrient agar plate and incubating at 37 °C for 24 h. The bacterial suspension density was adjusted to 0.5 M of McFarland Standard, which is equivalent to a bacterial suspension containing approximately 1 × 10^8^ CFU/mL. A dried surface of Mueller–Hinton agar was inoculated with each bacterium with a sterile swab. A film sample with a diameter of 15 mm was placed on the agar. The plate was incubated at 37 °C for 24 h. The diameter of the inhibition zone surrounding the film disc and the contact surface of the edible film with the agar surface were observed.

#### 4.5.8. Antioxidant Activity of the Edible Films

The measurement of the antioxidant activity of the edible films was based on a previously described method with modifications [6]. Twenty-five milligrams of film was dipped into 3 mL of methanol for 3 h to obtain a film extract solution. Then, 0.1 mL of the solution was added to 3.9 mL of 0.1 mM DPPH and incubated in the dark for 30 min before the absorbance was measured at 517 nm using a spectrophotometer. Methanol and DPPH solutions without the film extract were used as blanks, and the DPPH radical scavenging capacity was measured as follows:

(9) $$\%RSA=\frac{abs\;blanko-abs\;sample}{abs\;blanko}\times 100\%$$

## Figures and Tables

**Table 1:** Color properties and transparency at 600 nm of the films.

Film Samples	L*	a*	b*	ΔE*	Transparency (600 nm)
FLE 0%	86.19 ± 0.93 ^e^	−1.52 ± 0.08 ^e^	3.82 ± 0.21 ^a^	-	0.61 ± 0.02 ^a^
FLE 2.5%	82.21 ± 0.89 ^d^	−2.48 ± 0.22 ^d^	7.56 ± 0.12 ^b^	5.57 ± 0.66 ^a^	1.95 ± 0.24 ^b^
FLE 5%	80.27 ± 0.66 ^c^	−4.37 ± 0.29 ^c^	12.89 ± 0.59 ^c^	11.20 ± 0.87 ^b^	3.68 ± 0.01 ^c^
FLE 7.5%	77.47 ± 0.43 ^b^	−7.22 ± 0.26 ^b^	18.15 ± 0.27 ^d^	17.72 ± 0.32 ^c^	6.12 ± 0.28 ^d^
FLE 10%	71.14 ± 0.78 ^a^	−9.78 ± 0.18 ^a^	22.85 ± 0.22 ^e^	25.64 ± 0.34 ^d^	7.44 ± 0.23 ^e^

FLE: *Ficus carica* L. leaf extract; L*: brightness; a*: redness; b*: yellowness; ΔE*: total color difference. Significant differences (*p <* 0.05) are shown by different superscript letters in the columns.

**Table 2:** Physical properties of the films.

Film Samples	Thickness (mm)	Tensile Strength (MPa)	Elongation at Break (%)	Elastic Modulus (MPa)
FLE 0%	0.126 ± 0.01 ^a^	1.13 ± 0.20 ^a^	321.58 ± 15.33 ^ab^	0.35 ± 0.05 ^a^
FLE 2.5%	0.124 ± 0.01 ^a^	1.21 ± 0.21 ^a^	302.37 ± 17.97 ^a^	0.40 ± 0.09 ^ab^
FLE 5%	0.127 ± 0.01 ^a^	1.68 ± 0.21 ^b^	325.03 ± 25.58 ^ab^	0.52 ± 0.07 ^b^
FLE 7.5%	0.126 ± 0.01 ^a^	1.84 ± 0.25 ^b^	348.23 ± 9.28 ^bc^	0.53 ± 0.06 ^b^
FLE 10%	0.127 ± 0.01 ^a^	2.47 ± 0.28 ^c^	372.82 ± 14.89 ^c^	0.66 ± 0.05 ^c^

FLE: *Ficus carica* L. leaf extract. Significant differences (*p <* 0.05) are shown by different superscript letters in the columns.

**Table 3:** Physical properties of the films.

Film Samples	Solubility (%)	WVP × 10^−11^ (g/msPa)
FLE 0%	46.47 ± 0.28 ^e^	5.61 ± 0.25 ^c^
FLE 2.5%	42.80 ± 0.17 ^d^	5.04 ± 0.61 ^bc^
FLE 5%	40.86 ± 0.20 ^c^	4.89 ± 0.18 ^b^
FLE 7.5%	37.46 ± 0.29 ^b^	4.57 ± 0.27 ^ab^
FLE 10%	36.20 ± 0.09 ^a^	3.96 ± 0.18 ^a^

Significant differences (*p <* 0.05) are shown by different superscript letters in the columns.

**Table 4:** Antibacterial activity of the films.

Film Samples	Inhibition Zone (mm)	
	*S. aureus*	*E. coli*
FLE 0%	-	-
FLE 2.5%	18.64 ± 0.24 ^a^	-
FLE 5%	23.61 ± 0.12 ^b^	22.33 ± 0.22 ^a^
FLE 7.5%	25.69 ± 0.20 ^c^	23.17 ± 0.18 ^b^
FLE 10%	27.27 ± 0.15 ^d^	25.10 ± 0.28 ^c^

FLE: *Ficus carica* L. leaf extract. Significant differences (*p <* 0.05) are shown by different superscript letters in the columns.
